# Dog breeds and conformations in the UK in 2019: VetCompass canine demography and some consequent welfare implications

**DOI:** 10.1371/journal.pone.0288081

**Published:** 2023-07-26

**Authors:** Dan G. O’Neill, Kirsten M. McMillan, David B. Church, Dave C. Brodbelt

**Affiliations:** 1 Pathobiology and Population Sciences, The Royal Veterinary College, Hatfield, United Kingdom; 2 DataSEA (Science, Engineering & Analytics), Research Team, Strategy & Transformation, Dogs Trust, London, United Kingdom; 3 Clinical Science and Services, The Royal Veterinary College, Hatfield, United Kingdom; Sul Ross State University, UNITED STATES

## Abstract

**Introduction:**

Growing concerns over health and welfare impacts from extreme phenotypes in dogs have created an urgent need for reliable demographic information on the national breed structures of dogs.

**Methods:**

This study included all dogs under primary veterinary care in the UK during 2019 at practices participating in VetCompass. Demographic data on these dogs were analysed to report on the frequency of common breeds and also to report on conformation, bodyweight, sex and neuter associations with these breeds.

**Results:**

The study included 2,237,105 dogs under UK veterinary care in 2019. Overall, 69.4% (*n* = 1,551,462) were classified as purebred, 6.7% (149,308) as designer-crossbred and 24.0% (536,335) as nondesigner-crossbred. Across 800 unique breed names, the most frequent breeds at any age were nondesigner-crossbred (n = 536,335, 24.0%), Labrador Retriever (154,222, 6.9%) and Jack Russell Terrier (101,294, 4.5%). Among 229,624 (10.3%) dogs aged under one year, the most frequent breeds were nondesigner-crossbred (*n* = 45,995, 20.0%), French Bulldog (16,036, 7.0%) and Cockapoo (14,321, 6.2%). Overall, based on breed characteristics, 17.6% (395,739) were classified as brachycephalic, 43.1% (969,403) as mesaticephalic and 8.3% (186,320) as dolichocephalic. Of 1,551,336 dogs that were classifiable based on breed, 52.6% (815,673) were chondrodystrophic. Of 1,462,925 dogs that were classifiable, there were 54.6% (*n* = 798,426) short haired, 32.6% (476,883) medium haired and 12.8% (186,934) long haired. Of 1,547,653 dogs that were classifiable for ear carriage, 24.5% (*n* = 379,581) were erect, 28.1% (434,273) were semi-erect, 19.7% (305,475) were v-shaped drop and 27.7% (428,324) were pendulous. Overall, there was a 1.09:1.00 ratio of male (n = 1,163,512; 52.2%) to female dogs (n = 1,067,552; 47.8%).

**Conclusions:**

Health and welfare issues linked to popular breeds with extreme phenotypes suggest that there is much work to do to help owners to make more welfare-friendly decisions when choosing which type of dog to own.

## Introduction

Population ecology examines how and why populations change over time and space [1, 2]. It seeks to understand drivers of population abundance and sparsity, with a focus on assessing the influence of demographic parameters upon population structure as a result of underlying vital rates such as density, survival and recruitment [3–6]. Consequently, demography may be considered a ‘scaling’ tool: translating the fates of individuals into population-level outcomes [7]. Within the United Kingdom (UK), companion dogs, i.e., those not considered feral, free-roaming or stray, are the most popular domestic companion animal, with 31% of UK households reportedly owning a dog in 2022 [8]. In order to maximise health and welfare outcomes within our domestic canine population, researchers and policy makers require access to national demographic baselines with robust and generalisable data that permit reliable extrapolation of findings to inform future decision making [9]. In addition, these national demographic data would also allow evaluation of spatiotemporal patterns, point analyses, and benchmark comparisons [10]. Demographic insights are instrumental for the development of population-level strategic responses to changing or challenging conditions such as genetic bottlenecks, hereditary pathology, infectious disease and increasing popularity of breeds with extreme conformations. Despite these clear justifications for national demographic databases on dogs, there are limited published up-to-date and large-scale empirical data regarding domestic dog demographics within the UK [11, 12].

Dogs and man have a long joint history, with dogs being the first species domesticated by man and the only species known to share a domestic relationship with humans during the Pleistocene [13]. It is widely accepted that the grey wolf is the progenitor of the modern domestic dog but there is still debate on when and where this occurred [14]. Pinpointing temporal and geographical origins for domestic dogs has been challenging because of the genetic diversity within modern dogs as well as local extinctions of ancient wolves that leaves modern wolves as genetically distinct variants [15, 16]. However, the current evidence from skeletal remains attributable to present-day dog lineages combined with genetic estimates for separation between the ancestors of dogs and modern wolves suggest divergence and domestication between 40,000 and 14,000 years ago [17, 18]. Varying theories are still proposed on whether domestication of the dog occurred across multiple geographical areas with later merging of these populations or whether a single domestication occurred with later admixing by wolves from other regions [13, 15, 17, 19]. Either way, present-day diversity across more than 400 genetically distinct domestic dog breeds as defined by kennels clubs worldwide is a much more recent phenomenon, although many behavioural and physical phenotypes in domestic dogs still link variably to their original wolf progenitors [20–22]

During the mid-19^th^ century, the advent of breed clubs and the concept of breed standards provided structure for reproductive isolation of canine sub-populations, with the aim of ensuring breed differentiation and standardisation [23, 24]. This delineation between breeds was reinforced by new breeding practices such as the repeated use of popular sires, breeding to perpetuate desired physical or behavioural characteristics, promotion of the breed barrier rule, and population maintenance via inbreeding within closed familial lines [25–27]. As a result, many of the body conformational structures that define our present-day breeds were formalised in dogs within the last 150 years, such as ear carriage, skull shape, haircoat and body type [23, 24]. Whilst these selection practices successfully developed distinct breeds that were still originally highly specialised for specific functions such as herding, hunting and retrieving, they also provided a mechanism for unscrupulous/naive breeders to focus solely on selecting for extreme physical attributes that often were to the detriment of canine health, welfare, functionality, and behaviour [28].

A description of present-day UK-based dog demographics that includes information on important attributes such as sex, neuter status, age, breed and phenotype would offer analytical and inferential benefits to welfare, veterinary, educational, epidemiological and business stakeholders, amongst others. For example, national demographic structures are key determinants of inter-animal contact patterns and hence are critical for understanding infectious disease spread. As such, demographic insights would support the design and implementation of improved disease transmission control measures [29–33]. Demographic data would also improve our understanding of population composition and distribution, along with current welfare issues related to breed and conformation issues. Grounding these welfare issues within a population age structure would provide a mechanism to forecast prevalence within, or impact upon, future generations, allowing for the development of proactive, rather than reactive, response strategies to future demographic changes, e.g., designer-crossbred dogs [34]. Furthermore, these demographic data would provide much-needed insight into consumer demand because acquisition behaviours by owners ultimately translate into dog population dynamics. Recent research has suggested that, for some owners, physical appearance may be of greater importance in affecting decision making when acquiring a specified breed than the perceived risks of breed-associated diseases [35–38]. This issue is especially poignant because dog types selectively bred for extreme physical features such as flat faces, large heads, protruding eyes and folded skin are reported as predisposed to a wide range of conformation-associated disorders [39–41]. Despite this evidence base, consumer demand for dog types and breeds with extreme conformations continues to flourish [37].

Until recently, despite the recognised importance of having a good understanding of canine demography, it has been challenging to accurately describe the demography of the UK dog population. This was mainly due to a lack of representative and accessible data on UK dogs. Previous UK-based statistics on dog demography, such as those published by the Kennel Club [42] and the Pet Food Manufacturing Association [8], were widely cited and did provide useful contextual insight into certain population aspects. However, inherent systematic biases such as prerequisite pedigree status for Kennel Club data and limited sample size for Pet Food Manufacturing Association data reduced generalisability to the wider UK dog population. Furthermore, these earlier data sources often lacked methodological detail on how the data were collected and collated that further limited their wider application. Newer sources of information on dog breed frequencies such as ‘scraping’ data from online dog sales websites offer tantalising alternative views on breed popularity [43].

In an effort to address some of these limitations, the current study aimed to report the demography of dogs under primary veterinary care in 2019 across the UK using anonymised veterinary clinical data from the VetCompass Programme [44]. VetCompass is a welfare-focused programme at the Royal Veterinary College (RVC), London that collates de-identified electronic patient record (EPR) data from primary-care veterinary practices in the UK for epidemiological research [44]. An array of anonymised data fields covering demographic, clinical and therapeutic information are uploaded quasi real-time to a secure server at the RVC. Researchers access and explore these data using a single online portal. Currently, VetCompass collaborates with around 30% of UK veterinary practices (~1800 practices) to share data on over 26 million companion animals (including over 13 million dogs) that have supported over 115 peer-reviewed publications to date [45].

This study placed particular focus on reporting breed and conformational attributes of dogs in the UK. Detailed methodological data are provided showing the VetCompass system of using breed type information to classify dogs by a range of morphological criteria. The results of the current study could assist welfare scientists, breeders, veterinary practitioners and owners with a deeper understanding of the demographic structure of the wider UK dog population and can also support future work by other research groups that could apply these VetCompass breed classification systems and data on common breeds.

## Methods

The study population included all available dogs under primary veterinary care at clinics participating in the VetCompass Programme during 2019 [44]. Dogs under veterinary care were defined as having at least one EPR (free-text clinical note, treatment, or bodyweight) recorded during 2019. Canine demographic data fields available to VetCompass researchers and used in the current study included a unique animal identifier along with species, breed, date of birth, sex, neuter status, and bodyweight.

A cross-sectional study design was used to explore and report on demography and conformation in this population. Power calculations estimated that a sample of at least 366,193 dogs was needed to estimate the frequency of a breed that includes 0.1% of all dogs in a wider national UK population of 8 million dogs to a precision of 0.01% acceptable margin of error at 95% confidence level [46, 47]. All owners provided verbal opt-out informed consent. Ethics approval was obtained from the RVC Social Science Ethical Review Board (reference SR2018-1652).

Descriptive information on breed entered by the participating practices was cleaned and mapped to a VetCompass breed list derived and extended from the VeNom Coding breed list [48]. The breed information entered by the participating practices would generally have been based on a consensus between the owner(s) and the veterinary teams to agree on the most appropriate breed term to apply and could have been refined over time at repeated veterinary visits to reflect updated understanding. The current study used the latest available breed information terms for the analysis. Genetic ancestry testing or validation of pedigree records were not required for entry of breed information by the participating practices. In the context of this paper, the term ‘breed’ was broadly interpreted to include unique breed terms for each individual pure breed recognised by various kennel clubs and registries worldwide (collectively described as ‘purebred’ in the current study) and unique breed terms for each individual designer-crossbred (hybrid) type as defined below (collectively described as ‘designer-crossbred’ in the current study). Designer-crossbreds included types of dogs (included in the current paper as breeds) described with contrived portmanteau names generated from two or more purebred breed terms (e.g., Labradoodle) [34, 49]. All remaining dogs with non-missing breed information entered by the participating practices where the animal was described as a specified (e.g., lab x poodle, collie x) or unspecified (e.g., crossbred, mongrel) mix of breeds were included as ‘nondesigner-crossbred’ [50]. A *Purebred status* variable categorised the individual breeds as purebred, designer-crossbred or nondesigner-crossbred. A *Kennel Club breed group* variable classified breeds recognised by the UK Kennel Club into their relevant breed groups (Gundog, Hound, Pastoral, Terrier, Toy, Utility and Working) and all remaining types were classified as non-Kennel Club recognised [50]. Note that it was not possible to identify which individual dogs had a pedigree registered with the Kennel Club within the breeds that were recognised by the Kennel Club. Breeds were characterised by ear carriage based on pinnal phenotypes typically described for each breed [50–53]. The categories of ear carriage included erect (also known as prick or upright e.g., German Shepherd Dog), semi-erect (also known as cocked or semi-pricked e.g., Rough Collie), V-shaped drop (also known as folded e.g., Hungarian Vizsla), pendulous (also known as drop or pendant, e.g., Basset Hound) and unspecified. Based on information reviewed from multiple sources including several kennel club registries, previous publications, photographs sourced online, breeds were characterised where possible by skull shape (dolichocephalic, mesaticephalic, brachycephalic, unavailable), haircoat (short, medium, long, unavailable), chondrodystrophic (chondrodystrophic, non-chondrodystrophic, unavailable), spaniel-type status (spaniel, non-spaniel, unavailable), poodle-type status (poodle, non-poodle, unavailable) and Dachshund-type status (Dachshund, non-Dachshund, unavailable) for analysis. Among breeds that were categorised as brachycephalic, the degree of brachycephaly was estimated as mild, moderate or severe based on the typical characteristics of the breed [12] (S1 File). It should be noted that these categorisations were all achieved at the breed level and did not involve direct assessment of each dog at an individual level. These categorisations were mainly the responsibility of one author (DON) and therefore represent the current VetCompass classification system in the absence of another universal standard for breed classifications in dogs. Sex and neuter status were defined by the final available EPR value. For the purposes of the analysis, age (years) for each dog was defined at December 31, 2019. Adult bodyweight for each dog was defined as the median of all bodyweight (kg) values recorded for each dog after reaching 18 months old based on their recorded date of birth.

Following internal validity checking and data cleaning, analyses were conducted using Stata Version 16 (Stata Corporation). The median is reported rather than the mean to avoid assumptions of normality for continuous variables [54]. Proportions were reported with 95% confidence interval (CI) estimates derived from standard errors based on approximation to the binomial distribution [54]. Binary comparisons of continuous variables between subsets of dogs used the Mann-Whitney statistical test [54]. Statistical significance was set at *P* < 0.05.

## Results

### Breed frequency

The study included an overall population of 2,250,417 dogs at any age under veterinary care in 2019 within six veterinary groups participating in VetCompass across the UK. There were 13,312 (0.59%) dogs without any breed information recorded that were excluded from further analysis, leaving 2,237,105 dogs in the final analysis. The overall dataset included 800 unique dog breed names. The most frequent breeds at any age were nondesigner-crossbred (n = 536,335, 24.0% of all dogs), Labrador Retriever (154,222, 6.9%), Jack Russell Terrier (101,294, 4.5%), English Cocker Spaniel (96,824, 4.3%), Staffordshire Bull Terrier (93,883, 4.2%) and Chihuahua (80,609, 3.6%). The 10 most common breeds represented 59.56% of all dogs while the 20 most common breeds represented 75.93% of all dogs (Table 1 and S2 File).

**Table 1:** Descriptive statistics on the 40 most common dog breeds *at any age* under primary veterinary care in 2019 in the VetCompass™ Programme in the UK. *n* = 2,237,105.

Breed	Freq.	Percent	Age 31 Dec 2019—median	Adult bodyweight—median	Female adult bodyweight—median	Male adult bodyweight—median
Nondesigner-crossbred	536,335	24.0	5.4	12.7	12.2	13.0
Labrador Retriever	154,222	6.9	6.2	31.6	29.2	33.6
Jack Russell Terrier	101,294	4.5	8.3	7.9	7.3	8.4
English Cocker Spaniel	96,824	4.3	5.2	14.9	13.7	16.0
Staffordshire Bull Terrier	93,883	4.2	7.3	20.6	19.2	22.0
Chihuahua	80,609	3.6	4.2	3.8	3.6	4.0
Cockapoo	73,037	3.3	2.5	11.8	10.8	12.8
Shih-tzu	67,368	3.0	5.4	8.0	7.3	8.5
French Bulldog	66,997	3.0	2.0	12.9	11.8	13.8
Border Collie	61,802	2.8	6.4	21.0	19.5	22.4
Yorkshire Terrier	53,246	2.4	6.9	5.1	4.7	5.5
English Springer Spaniel	51,802	2.3	6.8	20.0	18.4	21.4
German Shepherd Dog	47,407	2.1	4.5	35.9	33.5	38.4
Pug	40,509	1.8	3.5	9.2	8.4	9.9
West Highland White Terrier	35,814	1.6	9.1	9.5	9.0	10.0
Cavalier King Charles Spaniel	35,240	1.6	6.9	10.4	9.8	11.0
Golden Retriever	27,491	1.2	5.5	33.5	31.6	35.4
Bichon Frise	25,162	1.1	6.8	8.3	7.7	8.7
Miniature Dachshund	24,831	1.1	2.7	6.4	6.0	6.8
Border Terrier	24,697	1.1	7.7	9.9	9.1	10.6
Lhasa Apso	24,553	1.1	6.7	8.3	7.6	8.9
British Bulldog	23,023	1.0	2.7	26.0	24.5	27.5
Labradoodle	21,802	1.0	4.5	24.7	23.0	26.5
Miniature Schnauzer	21,154	1.0	5.4	9.6	8.9	10.4
Beagle	20,229	0.9	4.8	18.0	16.6	19.3
Boxer	17,572	0.8	6.3	30.3	27.9	32.7
Husky	17,337	0.8	5.1	26.1	24.3	27.8
Lurcher	16,051	0.7	6.1	23.1	21.8	24.6
Pomeranian	14,844	0.7	3.3	5.4	5.0	5.8
Cavapoo	14,146	0.6	2.1	9.1	8.4	9.8
Rottweiler	13,203	0.6	4.3	42.3	39.2	45.5
Whippet	12,765	0.6	5.1	14.5	13.3	15.5
Greyhound	12,640	0.6	7.3	29.7	27.7	32.5
Patterdale Terrier	10,761	0.5	6.6	10.3	9.5	11.1
Sprocker	9,348	0.4	3.2	17.1	15.7	18.4
American Bulldog	8,633	0.4	2.1	37.5	35.0	40.0
Toy Poodle	8,601	0.4	5.1	5.1	4.6	5.4
Maltese	8,488	0.4	4.0	4.6	4.3	4.9
Hungarian Vizsla	7,199	0.3	3.8	25.2	23.3	27.2
Standard Dachshund	7,096	0.3	3.2	8.7	8.2	9.1

There were 229,624 dogs aged under one year in the study cohort, i.e., 10.2% of the population. The most frequent breeds aged under one year were nondesigner-crossbred (*n* = 45,995, 20.03%), French Bulldog (16,036, 6.98%), Cockapoo (14,321, 6.24%), Labrador Retriever (13,303, 5.79%), English Cocker Spaniel (10,766, 4.69%) and Chihuahua (9,637, 4.2%). The 10 most common breeds represented 58.79% of all dogs aged under one year while the 20 most common breeds represented 75.89% of all dogs aged under one year (Table 2).

**Table 2:** Descriptive statistics on the 20 most common dog breeds *aged under one year* under primary veterinary care in 2019 in the VetCompass™ Programme in the UK. *n* = 229,624.

Breed	Freq.	Percent
Nondesigner-crossbred	45,995	20.03
French Bulldog	16,036	6.98
Cockapoo	14,321	6.24
Labrador Retriever	13,303	5.79
English Cocker Spaniel	10,766	4.69
Chihuahua	9,637	4.20
Staffordshire Bull Terrier	6,909	3.01
German Shepherd Dog	6,322	2.75
Shih-tzu	6,067	2.64
Pug	5,633	2.45
Border Collie	5,329	2.32
Jack Russell Terrier	5,305	2.31
Miniature Dachshund	5,267	2.29
British Bulldog	3,987	1.74
Cavapoo	3,786	1.65
English Springer Spaniel	3,677	1.60
Yorkshire Terrier	3,502	1.53
Golden Retriever	3,200	1.39
Labradoodle	2,676	1.17
Cavalier King Charles Spaniel	2,543	1.11

There were 445,884 dogs aged over 10 years in the study, i.e., 19.9% of the population. The most frequent breeds aged over 10 years were nondesigner-crossbred (*n* = 105,360, 23.63%), Jack Russell Terrier (38,204, 8.57%), Labrador Retriever (38,074, 8.54%), Staffordshire Bull Terrier (28,816, 6.46%), English Cocker Spaniel (18,390, 4.12%) and Border Collie (17,077, 3.83%). The 10 most common breeds represented 67.93% of all dogs aged over 10 years while the 20 most common breeds represented 81.55% of all dogs aged over 10 years (Table 3).

**Table 3:** Descriptive statistics on the 20 most common dog breeds *aged over 10 years* under primary veterinary care in 2019 in the VetCompass™ Programme in the UK. *n* = 445,884.

Breed	Freq.	Percent
Nondesigner-crossbred	105,360	23.63
Jack Russell Terrier	38,204	8.57
Labrador Retriever	38,074	8.54
Staffordshire Bull Terrier	28,816	6.46
English Cocker Spaniel	18,390	4.12
Border Collie	17,077	3.83
West Highland White Terrier	15,634	3.51
Yorkshire Terrier	15,023	3.37
English Springer Spaniel	14,500	3.25
Shih-tzu	11,825	2.65
Cavalier King Charles Spaniel	8,467	1.90
Border Terrier	8,292	1.86
German Shepherd Dog	7,373	1.65
Chihuahua	7,154	1.60
Golden Retriever	6,368	1.43
Lhasa Apso	6,296	1.41
Bichon Frise	5,593	1.25
Miniature Schnauzer	3,878	0.87
Lurcher	3,780	0.85
Greyhound	3,513	0.79

### Purebred status

Overall, 69.4% (*n* = 1,551,462) of dogs were classified as purebred with 6.7% (149,308) classified as designer-crossbred and 24.0% (536,335) as nondesigner-crossbred. Designer-crossbred dogs (median age 3.02 years, interquartile range [IQR] 1.39–5.63, range 0.00–24.33) were statistically significantly younger than nondesigner-crossbred dogs (median 5.40, IQR 2.51–8.90, range 0.00–24.95) (*P* < 0.001) and purebred dogs (median 5.47, IQR 2.31–9.28, range 0.00–24.98) (*P* < 0.001). Designer-crossbred dogs (median adult bodyweight 13.40 kg, IQR 10.22–19.00, range 2.15–57.00) were statistically significantly heavier than nondesigner-crossbred dogs (median 12.65 kg, IQR 28.22–22.16, range 4.00–41.65) (*P* < 0.001) and lighter than purebred dogs (median 14.25 kg, IQR 8.30–25.90, range 0.38–106.00) (*P* < 0.001) (Table 4). Proportional purebred status varied between the young and old dogs (P < 0.001), with designer-crossbred comprising 11.2% of dogs aged under one year compared with 2.2% of dogs aged over 10 years (Table 5).

**Table 4:** Descriptive statistics for major groupings of dogs under primary veterinary care in 2019 in the VetCompass™ Programme in the UK. *n* = 2,237,105.

Variable	Category	No.	%	Median age (31 Dec 2019)	Median adult bodyweight (≥ 18 mts)	Female—median adult bodyweight	Male—median adult bodyweight
Purebred status	Nondesigner-crossbred	536,335	24.0	5.4	12.7	12.2	13.0
	Designer-crossbred	149,308	6.7	3.0	13.4	12.2	14.4
	Purebred	1,551,462	69.4	5.5	14.3	13.5	15.0
Skull-shape	Brachycephalic	395,739	17.6	3.9	9.1	8.4	9.7
	Mesaticephalic	969,403	43.1	6.4	17.6	16.5	18.8
	Dolichocephalic	186,320	8.3	4.8	19.5	20.6	18.4
	Not applicable	698,955	31.1	4.7	12.9	12.2	13.5
Breed recognised by The Kennel Club (KC)	Not recognised	726,094	32.5	4.7	13.0	12.3	13.5
	Recognised	1,511,011	67.5	5.5	14.3	13.5	15.0
KC breed group	Not KC recognised	726,094	32.5	4.7	13.0	12.3	13.5
	Gundog	372,428	16.7	5.8	26.0	24.7	27.9
	Hound	97,526	4.4	4.6	15.5	15.0	16.1
	Pastoral	127,422	5.7	5.6	25.0	23.5	26.0
	Terrier	292,638	13.1	7.9	10.3	9.7	10.7
	Toy	278,725	12.5	5.0	6.2	5.7	6.6
	Utility	264,749	11.8	3.9	10.0	9.3	10.6
	Working	77,523	3.5	4.9	35.4	33.1	37.6
Sex	Female	1,067,552	47.85	5.34	12.96	~	~
	Male	1,163,512	52.15	5.19	14.30	~	~
	Hermaphrodite	1	0.00	1.16	~	~	~
Neuter status	Entire	1,255,098	56.26	3.21	13.40	12.20	13.60
	Neutered	975,966	43.74	7.45	14.00	14.35	14.30
Sex-Neuter	Female entire	589,600	26.43	3.13	12.20	~	~
	Female neuter	477,952	21.42	7.70	13.60	~	~
	Male entire	665,498	29.83	3.27	14.35	~	~
	Male neuter	498,014	22.32	7.22	14.30	~	~

**Table 5:** Proportions of categories aged under one year (*n* = 229,624) and aged over 10 years (*n* = 445,884) within major groupings of dogs under primary veterinary care in 2019 in the VetCompass™ Programme in the UK.

Variable	Category	Under 1 year		Over 10 years	
	Category	No.	%	No.	%
Purebred status	Nondesigner-crossbred	45,995	20.0	105,360	23.6
	Designer-crossbred	25,727	11.2	9,573	2.2
	Purebred	157,902	68.8	330,951	74.2
Skull-shape	Brachycephalic	54,158	34.3	46,042	13.9
	Mesaticephalic	80,824	51.2	251,113	75.9
	Dolichocephalic	22,920	14.5	33,796	10.2
Breed recognised by The Kennel Club (KC)	Not recognised	78,754	34.3	121,612	27.3
	Recognised	150,870	65.7	324,272	72.7
KC breed group	Gundog	35,098	23.3	85,473	26.4
	Hound	12,426	8.2	15,933	4.9
	Pastoral	13,343	8.8	28,993	8.9
	Terrier	17,085	11.3	101,930	31.4
	Toy	28,118	18.6	45,112	13.9
	Utility	35,929	23.8	36,142	11.2
	Working	8,871	5.9	10,689	3.3

### Kennel Club

Of 2,237,105 dogs with breed (including crossbreds) information available, 67.54% (n = 1,511,011) were of a breed recognised by the UK Kennel Club (Kennel Club, 2022a) and 32.46% (726,094) were of a breed not recognised by The Kennel Club. It should be noted that it was not possible to ascertain which individual dogs within the breeds regcognised by The Kennel Club were actually registered with The Kennel Club. Among the breeds recognised by The Kennel Club, the most common breed groups were Gundog (n = 372,428, 24.65%), Terrier (292,638, n = 19.37%) and Toy (278,725, 18.45%) (Table 4). Kennel Club recognised breeds comprised 65.7% of dogs aged under one year compared and 72.7% of dogs aged over 10 years (Table 5).

### Breed-family types

There were 1,700,770 (76.03) dogs that were recorded as either a purebred or a designer breed. Of these, 11.88% (*n* = 202,015) were purebred Spaniel types, 5.80% (98,640) were Spaniel-designer crosses and 82.32% (1,400,115) were non-Spaniel types. There were 1.24% (*n* = 21,055) purebred Poodle, 6.70% (113,933) Poodle designer crosses and 92.06% (1,565,782) non-Poodle types. There were 2.59% (*n* = 44,099) purebred Dachshund types, < 0.01% (36) Dachshund-designer crosses and 97.40% (1,656,635) non-Dachshund types.

### Skull length

Overall, 17.59% (*n* = 395,739) of dogs were classified by breed as brachycephalic, 43.08% (969,403) as mesaticephalic and 8.28% (186,320) as dolichocephalic (Table 4). Among the breeds with brachycephaly that were gradable by severity, 11.96% (n = 47,313) were graded as mildly brachycephalic, 34.15% (135,154) were moderately brachycephalic and 53.89% (213,272) were severely brachycephalic. Brachycephalic dogs (median age 3.92 years, IQR 1.67–7.21, range 0.00–24.98) were statistically significantly younger than mesaticephalic dogs (median 6.38, IQR 2.81–10.00, range 0.00–24.79) (*P* < 0.001) and dolichocephalic dogs (median 4.82, IQR 1.97–8.64, range 0.00–24.84) (*P* < 0.001). Brachycephalic dogs (median adult bodyweight 9.10 kg, IQR 6.40–12.60, range 1.50–106.00) were statistically significantly lighter than mesaticephalic dogs (median 17.60 kg, IQR 9.50–26.80, range 0.38–97.50) (*P* < 0.001) and dolichocephalic dogs (median 19.52 kg, IQR 8.30–32.50, range 2.00–88.00) (*P* < 0.001). Proportional skull shape varied widely between the young and older dogs (P < 0.001), with mesaticephalic dogs comprising 51.2% of dogs aged under one year compared with 75.9% of dogs aged over 10 years while conversely, brachycephalic dogs comprised 34.3% of dogs aged under one year compared with 13.9% of dogs aged over 10 years (Table 5).

### Other conformation characteristics

Of 1,551,336 dogs that could be classified by their breed according to their chondrodystrophy status, 52.58% (*n* = 815,673) were chondrodystrophic and 47.42% (735,663) were non-chondrodystrophic. Of 1,462,925 dogs that could be classified according to their haircoat, 54.58% (*n* = 798,426) were short haired, 32.60% (476,883) were medium haired, 12.78% (186,934) were long haired and 0.05% (682) were hairless. Of 1,547,653 dogs that could be classified by the ear carriage conformation, 24.53% (*n* = 379,581) were erect, 28.06% (434,273) were semi-erect, 19.74% (305,475) were v-shaped drop and 27.68% (428,324) were pendulous (Table 4).

### Sex

**Fig 1:**
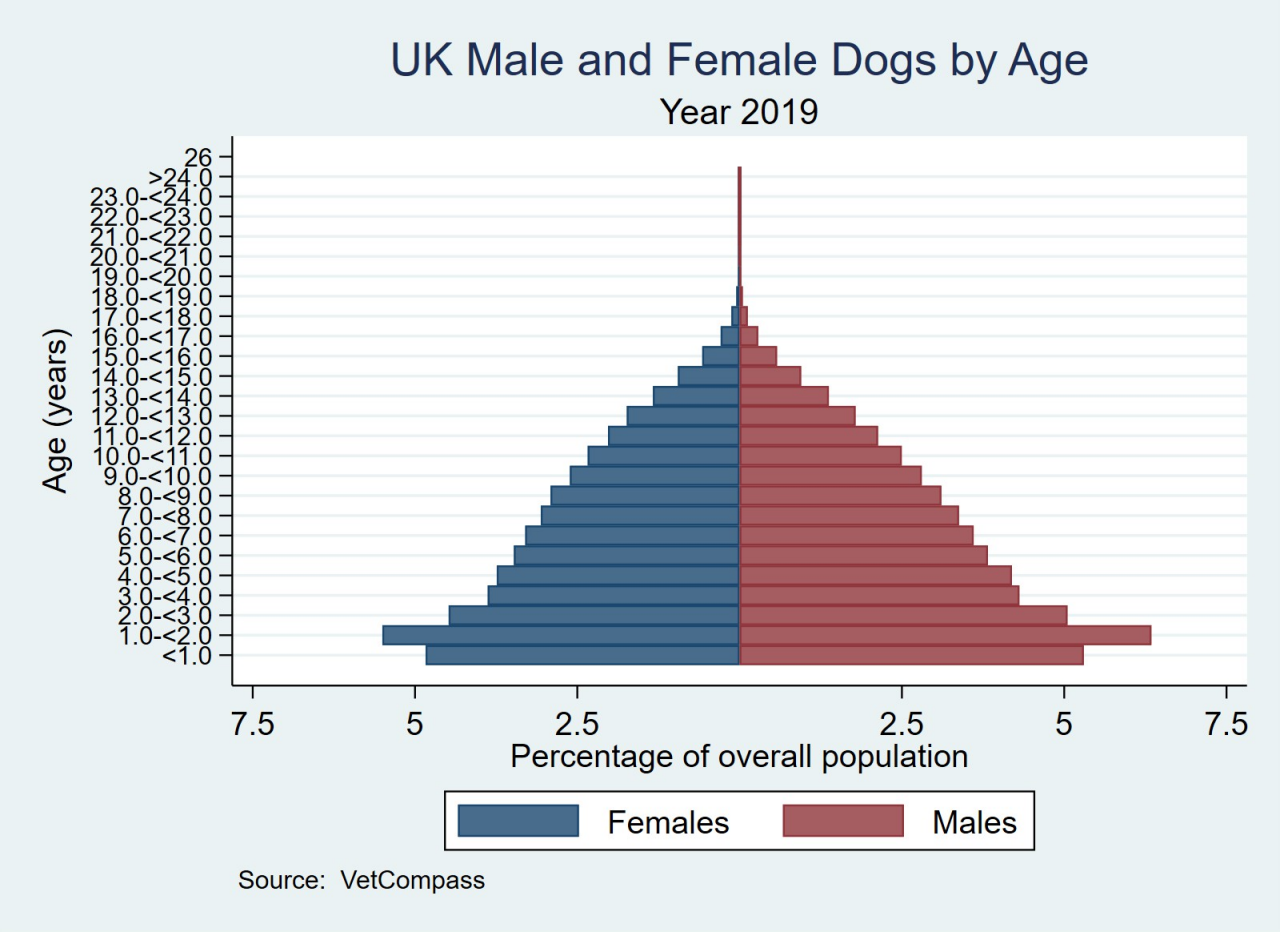
Age distribution by sex in dogs under primary veterinary care in 2019 in the VetCompass™ Programme in the UK. *n* = 2,215,462.

Overall, there were 2,231,065 dogs with sex status recorded. There was a 1.09:1.00 ratio of male (n = 1,163,512; 52.15%) to female dogs (n = 1,067,552; 47.85%), and one animal recorded as hermaphrodite. The median age of females (median 5.34 years, IQR 2.31–9.11, range 0.00–24.91) was statistically older than for males (median 5.19, IQR 2.24–8.89, range 0.00–24.98) (*P* < 0.001). The median adult bodyweight of females (median 12.96 kg, IQR 7.87–23.20, range 0.38–90.25) was lighter than for males (median 14.30 kg, IQR 8.95–25.70, 1.55–106.00) (*P* < 0.001) (Fig 1).

### Neuter status

**Fig 2:**
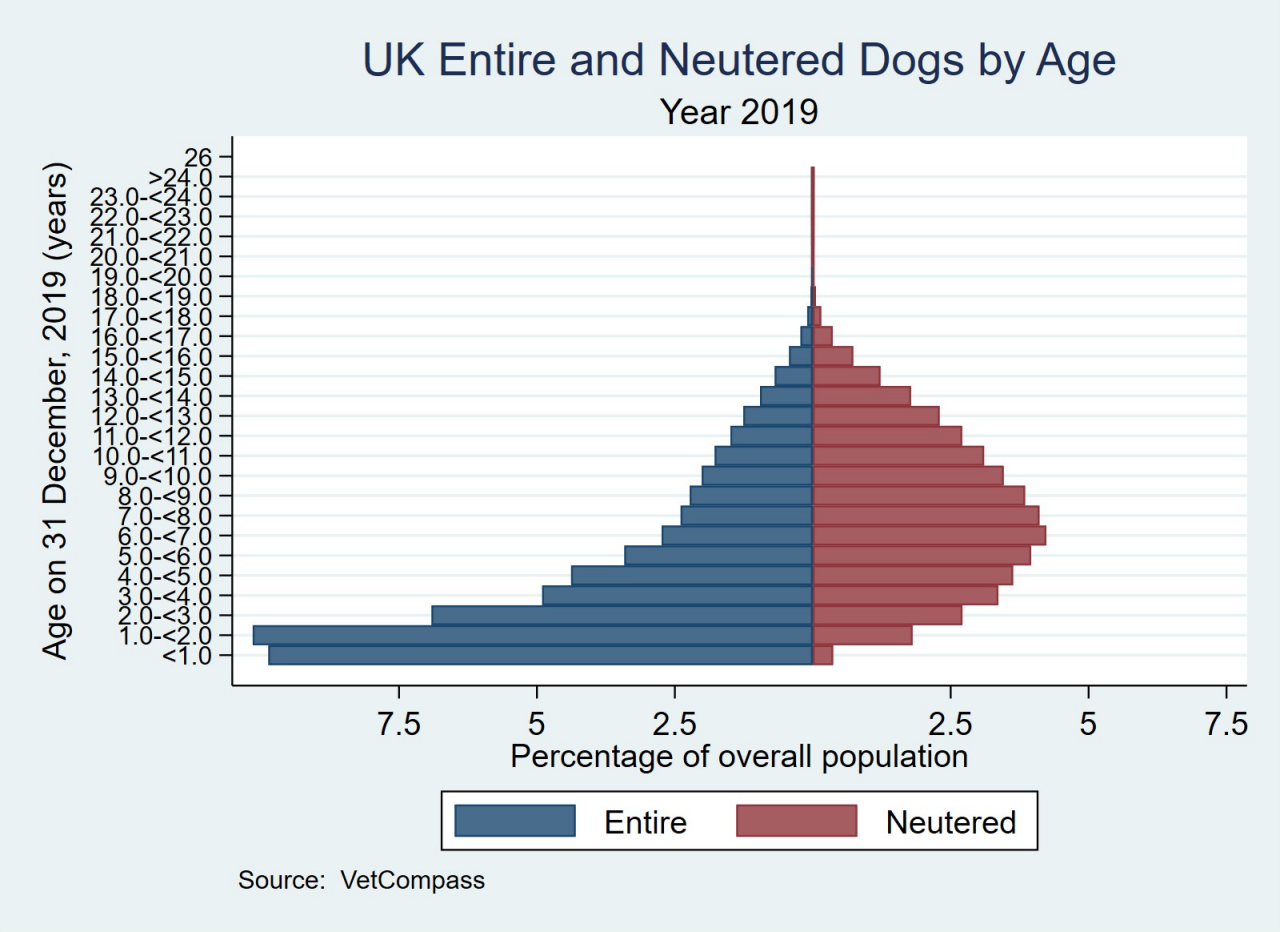
Age distribution by neutered status in dogs under primary veterinary care in 2019 in the VetCompass™ Programme in the UK. *n* = 2,215,462.

Of the 2,231,064 dogs with neuter status recorded, there were 975,966 (43.74%) animals recorded as neutered on their final available clinical record. Across all ages, female dogs (477,952/1,067,552, 44.77%) were statistically significantly more likely to be neutered than males (498,014/1,163,512, 42.80%) (P < 0.001). Among dogs aged under one-year, female dogs (3,694/108,220, 3.41%) were statistically significantly less likely to be neutered than males (4,497/118,612, 3.79%) (P < 0.001). Among dogs aged over 10 years, female dogs (145,759/221,563, 65.79%) were statistically significantly more likely to be neutered than males (132,297/225,654, 58.63%) (P < 0.001). Across all ages, proportional neutering differed between non-designer crossbred (260,403/531,343, 49.01%), designer crossbred (60.095/148,184, 40.55%) and purebred (652,850/1,538,781, 42.43%) dogs (P < 0.001). In non-designer crossbred dogs across all ages, female dogs (127,261/257,873, 49.35%) were statistically significantly more likely to be neutered than males (133,142/273,470, 48.69%) (P < 0.001). In designer crossbred dogs across all ages, female dogs (29,128/70,824, 41.13%) were statistically significantly more likely to be neutered than males (30,967/77,360, 40.03%) (P < 0.001). In purebred dogs across all ages, female dogs (320,261/731,400, 43.79%) were statistically significantly more likely to be neutered than males (332,589/807,381, 41.19%) (P < 0.001). Neutered animals (median 7.45 years, IQR 4.74–10.46, 0.00–24.98) were statistically significantly older than entire animals (median 3.21, IQR 1.32–6.85, 0.00–24.95) (*P* < 0.001) (Fig 2). The median adult bodyweight of neutered animals (median 14.00 kg, IQR 8.70–24.50, range 1.60–106.00) was heavier than for entire animals (median 13.40 kg, IQR 8.10–24.30, 0.38–100.00) (*P* < 0.001).

## Discussion

Population dynamics within the UK dog population are inherently tied to human ecology and behaviour. As such, the demographic landscape of dogs described here has been moulded, in part, by social [55], cultural [56, 57], economic [58, 59], technological [60] and legislative change to the canine ‘marketplace’ [61]. These broader drivers overlay the intrinsic behavioural and motivational drivers at the individual human level that sum together to translate into the overall population-level structure of the national canine demography [34, 37, 38, 62]. Our study reports breed and conformational characteristics within the UK dog population in 2019, whilst drilling further down into demographic attribute comparisons between sub-populations, such as age and bodyweight differences between skull-shape categories. In this discussion, we (1) explore the trajectory of breed popularity over the past 10–15 years, including purebred, nondesigner-crossbredand designer-crossbred groups; (2) highlight evidence for an increasing popularity of designer-crossbreds (especially poodle-crosses) over time; and (3) propose a likely future worsening health and welfare crisis for dogs overall related to extreme conformations and hereditary diseases if current market forces are not altered to prioritise innate health as a driving factor in dog purchasing decisions in the future [63]. Furthermore, these data provide a much-needed baseline demographic comparator to enable generalisability via VetCompass for other national and international research projects with smaller or more biased datasets to the wider UK dog population [64, 65].

The modern companion dog has become the most phenotypically diverse domestic species on the planet, featuring hundreds of recognisable breeds that each represents a unique collage of morphological, behavioural, and physiological traits [24, 66, 67]. Despite this spectacular diversity across the extant dog breeds, our data report remarkably consistent preferences for a limited list of popular dog breeds as companion dogs, with the ten most common breeds representing nearly 60% of all dogs in the UK. Furthermore, the high proportion of dogs within our UK data that represent breeds recognised by The Kennel Club suggests that the influence of The Kennel Club and dog shows such as Crufts upon consumer behaviour goes far beyond the limited subset of just 30% of UK dogs that are estimated to be registered with the KC [42, 68, 69].

Selection pressures towards exaggerated and extreme physical traits that are associated with high levels of conformation-related and hereditary pathology has led to major discussion and rethinking, especially over the past 15 years, around the ethics and welfare implications of breed as a concept for dogs [62, 70–75]. However, despite a large evidence base on serious health and welfare concerns, breeding towards extreme conformations as laid out in breed standards that were often arbitrarily drafted over a century ago continues to be implemented to gain a competitive advantage in dogs used for showing [24, 76] or to meet ongoing consumer demand for quirky or unique physical attributes in dogs kept as companion animals (Sandøe et al. 2017). Sadly it remains a reality that so long as the market forces of supply and demand for dog breeds continue to be driven more by human whims rather than by prioritising the needs of dogs to have good innate health, then it will remain challenging to shift the overall canine breed structure in any meaningful way away from breeds and types of dogs with high risks of breed-associated and conformation-associated ill-health [63, 77, 78].

Reliable data on population dynamics and health are a key component of effective canine health and welfare surveillance [79, 80] and can also have financial, legal and emotional impacts on owners, and may ultimately also impact the probability of canine euthanasia and relinquishment [81]. Despite an increasing scientific evidence-base outlining a range of serious health challenges facing dog breeds with brachycephaly [12], the ownership of certain brachycephalic breeds such as the Pug, French Bulldog and English Bulldog has increased dramatically over the past decade both in the UK [42] and internationally [82]. This pattern of proportionally high kennel club registrations for pedigree dogs with brachycephaly is mirrored within our data that reflects the wider UK dog population, with nearly 35% of dogs aged under one year in 2019 in the current study categorised as brachycephalic. Consequently, if these popularity trends continue, we predict a looming health and welfare crisis as this cohort of dogs with brachycephaly ages from early life into later life when the risk rises dramatically for many brachycephaly-associated disorders that are age-related such as BOAS [83], corneal ulceration [84] and skin fold dermatitis [85]. This potential worsening health and welfare crisis related to brachycephaly in dogs in the future has major implications not only for the individual animals affected but also for the broader provision of veterinary health care nationally as the veterinary profession will come under even greater pressure to provide for these increasing health care demands.

An additional new phenomenon in breed distributions identified in the current work is the substantial increase in designer-crossbred breed types [34]. Designer-crossbred breed types have emerged particularly over the last 15 years, with designer-crossbreds comprising only 2.2% of dogs aged over 10 years compared to 11.2% among dogs aged under one year. The longer-term welfare impacts from this designer-crossbred demographic shift remains largely unknown as there is a paucity of evidence to date on their relative health and disease predispositions and work is now urgently needed to explore these questions and fill these information gaps.

The population sex ratio in the current study was skewed towards males (1.09 male-to-female ratio) in line with many previous reports [11, 86, 87]. The adult sex ratio is a central concept of population demography and may reflect differential contributions emerging from differences in sex ratio at birth, sex differences in juvenile or adult dog mortality that may be driven by differential sex differences in maturation times, health and behaviours [88–90]. However, it is also possible that the adult sex ratio differences reported for dogs in the current study and consistently in other studies may reflect biases that are intrinsic to how these study data were collected. For example, the current study relies on data on dogs that are presented for primary veterinary care. One contribution to a male-skew in presentation for primary veterinary care could be a greater frequency of male neutering compared to female neutering, perhaps driven by a public perception regarding the relative ease and lower cost of neutering males that is reinforced by owner concerns regarding undesirable behaviours in entire males [11, 90]. However, research based within the UK, Ireland, and USA suggests that this is unlikely to be a major factor because veterinary surgeons are more likely to recommend neutering of female dogs than male dogs [11, 87, 91–94]. Another possibility to explain proportionally higher males in the current primary care veterinary data would be an intrinsically poorer heath profile of male dogs whereby these dogs were more likely to be presented for veterinary care than females. However, recent work comparing the health status of male and female dogs has again largely ruled out this explanation by showing no difference in the probability of having at least one disorder recorded between male and female dogs under primary veterinary care in the UK [87]. Given that female dogs are reported to live longer than male dogs [95], this suggests that the male-skewed sex ratio reported in the current study and elsewhere reflects increased recruitment of males via higher immigration rates into the UK [96], male-biased birth sex ratios, and/or consumer preference [56, 97–101]. Despite concerns regarding long-term social and population consequences of adult sex ratio biases that have been repeatedly raised within human [102–105] and non-human populations [106–108], our understanding of the impact of adult sex ratio on population characteristics, particularly of the magnitude reported here, is still limited. Future research could focus on examining demographic mechanisms that give rise this male-skew within UK dogs, along with assessing the preference of current/prospective dog owners for female versus male dogs, and how such acquisition behaviours feeds back into population dynamics.

The influence of demographic change in dogs upon the spread of zoonotic infection should not be under-estimated. Demographic characteristics of dog populations have been shown to greatly affect the transmission and maintenance of a range of zoonotic pathogenic agents [29, 32, 33]. Such knowledge can influence the planning, implementation and monitoring of infectious disease control programs [109], especially with regards to vaccination campaigns by supporting decision-making on the quantity of vaccines needed, the frequency of administration and the optimal populations to target [30, 31, 110, 111]. Thus, knowledge of population dynamics is key in the surveillance and monitoring of canines as a zoonotic disease vector and this One Health awareness is likely to become more and more critical as new and emerging zoonotic disorders involving dogs develop in the UK and elsewhere [32, 80, 112, 113].

In addition to providing information directly on the demography of dogs in the UK, the current study also provides a demographic resource that can be used by other researchers and research groups to ground their own research. Many research projects collect information on numerator cases, e.g., dogs recorded with a clinical condition such as Alabama rot, but do not have ready access to a denominator sampling frame for the underlying population of dogs from which these affected dogs (i.e. cases) was drawn. The consequence of this is that such studies with only cases are limited to reporting descriptive information on these cases or to reporting risk factor analysis just within the cases (e.g., probability of outcomes in male versus female cases) themselves but are unable to report demographic risk factor results that help to explain why cases became cases in the first place. However, the detailed breed demography of UK dogs provided by the current study can enable other researchers and research groups to extend their own analyses to report on risk factors relative to the wider UK dog population. This concept of using a VetCompass denominator demographic population to underpin research on cases identified from other datasets has already been validated on disorders including Alabama rot [65] and leptospirosis [114].

This study had some limitations. Although a high proportion of owned companion animals tend to receive veterinary care in countries with developed pet industries, it is estimated that just 77% of owned dogs in the UK are formally registered with a veterinary practice [46]. Therefore, although Big Data resources such as VetCompass may provide demographic data on large numbers of dogs in the UK, it is possible that intrinsic demographic differences between dogs registered and unregistered for veterinary care still limit the generalisation of the current results to the total UK dog population. In contrast, The Kennel Club publishes numbers on all UK dogs registered with The Kennel Club on a quarterly basis but these data are limited to only those 222 breeds that are recognised by The Kennel Club and within these breeds, only to the subset of these dogs that are pedigreed [115]. Another source of UK dog demographic data is provided by the annual rolling national dog population estimate from the Pet Food Manufacturing Association (PMFA), the most recent of which reported 13.0 million dogs in the UK for 2022 with 31% of UK households having at least one dog [8]. However, however the methods of the PFMA survey are limited by a relatively small sample of around 9,000 UK households per year, equating to a sampling rate of around only 0.04% of the estimated 24.8 million households estimated in England and Wales in 2021 [116].

This paper aimed primarily to report on breed and conformational factors in UK dogs. Although information on neuter status was provided, this was not a primary focus of the paper. Breed and conformation status are fixed over time in individual dogs and therefore a cross-sectional analysis such as the current study is appropriate. However, neuter status is a time-varying parameter (i.e., an individual dog can be both entire and neutered at different points in their life) and therefore a study with a primary focus on exploring neuter status would require a cohort design with animals followed over time to identify the age at neutering. Unfortunately, information on the dates of neutering were not available for the current analysis and so the results on neutering given here should be interpreted with caution.

The current study relied on the breed status information recorded in the veterinary clinical records. Although these breed terms reflected the sum of the insights of the owners and the relevant veterinary teams and could be updated over time in the clinical records to improve accuracy, it is still possible that some misclassification on the precise breed existed. The breed status of each dog was not individually validated using genetic ancestry tests, cross-referencing to kennel club registries or using photographs. The current study did not aim to link the VetCompass and Kennel Club datasets and therefore was unable to identify the subset of VetCompass dogs that were registered with The Kennel Club. The classifications within breeds for phenotypic characteristics such as ear carriage, hair coat and skull shape were derived based on typically expected values for the breed but it is possible that these values did not apply equally to each individual dog within each breed. The growing phenomenon of designer-crossbred dog breeds in the UK means that many of the breeds listed in the current study may not be classically considered as formal breeds according to definitions developed over the past century by kennel clubs [50]. Breed distributions for dogs aged under 1 and over 10 years reflect effects from both breed popularity and longevity; these longevity effects are likely to have substantial impact on the breed distribution for dogs aged over 10 years in the current study because of the widely differing longevity reported across breed in dogs [95].

## Conclusions

In conclusion, this analysis of over 2 million dogs under primary veterinary care in the UK during 2019 has identified that the most common breeds overall were the nondesigner-crossbred, Labrador Retriever and Jack Russell Terrier. However, changing preferences for extreme breeds and the emergence of new designer-crossbred breeds are reflected by differing breed profiles in dogs aged under one year where the most common breeds were nondesigner-crossbred, French Bulldog and Cockapoo. Brachycephaly was shown to be a highly popular phenotype among UK dog owners, with 17.6% of UK dogs representing a breed with brachycephaly, raising substantial questions about the canine welfare impact from our collective breed selection choices.

## Supporting information

S1 File VetCompass metadata linking UK dog breed terms to a range of demographic and phenotypic descriptors. https://rvc-repository.worktribe.com/output/1596183.
(TXT)

S2 File Frequency of all dog breeds under primary veterinary care in the VetCompass™ Programme in the UK. *n* = 2,237,105. https://rvc-repository.worktribe.com/output/1596183.
(TXT)

## Abbreviations

CI: confidence interval
EPR: electronic patient record
IQR: interquartile range
IRR: incidence risk ratio
KC: The Kennel Club
OR: odds ratio
PMFA: Pet Food Manufacturing Association
RVC: Royal Veterinary College
